# Linguistic Rule Generalisation Creates the Same Distributional Structure That Feeds It

**DOI:** 10.1162/OPMI.a.253

**Published:** 2025-11-10

**Authors:** Elizabeth Pankratz, Jennifer Culbertson, Simon Kirby

**Affiliations:** Psychology, University of Edinburgh, Edinburgh, UK; Centre for Language Evolution, University of Edinburgh, Edinburgh, UK

**Keywords:** rule generalisation, frequency distributions, artificial language learning, urn model, Bayesian reasoning

## Abstract

Part of language’s great expressivity comes from its users creating new forms by applying familiar rules to novel items. But linguistic rules aren’t all created equal—some are more readily generalisable than others. In this paper, we focus on how rule generalisation is affected by certain properties of frequency distributions. In an artificial language learning experiment that asks adult learners to generalise using one of two suffixes, we find that they probability-match their input but slightly prefer whichever suffix they encountered with more low-frequency stems. Then with an urn model of learning, we show that previous explanations of generalisation that focus only on a distribution’s type count or its skew fail to capture participants’ behaviour—only the low-frequency preference yields convergent results. We model learners’ behaviour in terms of rational Bayesian inference about how likely a rule is to apply to more word types than somebody has already encountered. Overall, we suggest that linguistic rule generalisation is a self-sustaining process: by creating novel and therefore low-frequency items, rule generalisation produces the very same distributional structure that feeds it.

## INTRODUCTION

A core feature of human language is how readily its parts can be combined to create new forms. But to create those forms, people need to identify which rules can be applied in novel ways, and which cannot. How does a language user know whether they can generalise a rule to a novel item to create a form they’ve never seen before?

Researchers have uncovered factors such as the phonology (e.g., Orsolini et al., 1998) and the semantics (e.g., Watson et al., 2021) of previously-seen items. For example, Orsolini et al. (1998) describe how Italian children allocate novel words to the putatively low-productivity conjugation class III, generalising the inflectional rules of that class to those stems, if the phonology of the novel words matches existing Class III items. And Watson et al. (2021) show that the semantics of novel coinages—novel applications of a rule—tends to match the semantics of familiar items that have lower frequency, rather than the semantics of the high-frequency items. The central role of low-frequency items is a theme that carries into the present paper. Here, we use artificial language learning and computational modelling to understand how language users draw on the distributional structure of their input to decide which of two possible patterns, or rules, should be generalised to novel items.

We draw specifically on previous explanations of how rule generalisation is facilitated by a sample’s type count, on the one hand, and its skew, on the other. In what follows, we review previous findings about type count (Section Distributions Over More Types Facilitate Generalisation) and skew (Section Skewed Distributions Facilitate Generalisation), along with other prominent distribution-based measures of a rule’s generalisability (Section Other Distributional Accounts of Rule Productivity). Then we offer a proposal that encompasses and unifies existing findings: that people prefer to generalise the rule that they’ve encountered with a greater number of rare types (Section The Present Proposal).

### Distributions Over More Types Facilitate Generalisation

Many linguists have observed that the more items a rule has been applied to, the more generalisable that rule tends to be (e.g., Baayen, 2001; Bybee, 2008; de Jong et al., 2000; Gómez, 2002; Yang, 2016). We refer here to the number of distinct items a rule applies to as a rule’s “type count”, following Baayen (2001); it has also been known as (inter alia) “family size” (de Jong et al., 2000), “set size” (Gómez, 2002), and “contextual diversity” (Tamminen et al., 2015).

That a greater type count facilitates rule generalisation has been observed experimentally across a range of morphosyntactic structures. It holds for rules relating to the order of words within phrases and sentences (Valian & Coulson, 1988), non-adjacent dependencies of the shape *aXb* (Gómez, 2002), adjacent dependencies like stem–suffix combinations (Experiment 2 in Tamminen et al., 2015), and word-internal patterns of syllable repetition (Radulescu et al., 2020).

This effect tends to be explained in terms of variability, which is known to aid generalisation (Raviv et al., 2022). The more different types that are encountered in a particular context, the more the invariable parts of that context stand out (Gómez, 2002; see also the information-theoretical formalisation in Radulescu et al., 2020).

### Skewed Distributions Facilitate Generalisation

In another strand of research, Casenhiser and Goldberg (2005) and Goldberg et al. (2004) focus not on the number of distinct items but on their relative token frequencies.

To illustrate: the sample [*cat, cat, dog, dog, sheep, sheep*] contains three different types, each appearing an equal number of times. Counting up how many tokens of each type this sample contains would produce a frequency distribution that is uniform. A different sample, [*cat, cat, cat, cat, dog, sheep*] contains mostly tokens of one particular type, with fewer tokens of all the others. Tallying the tokens in this sample would yield a frequency distribution that is skewed.

Goldberg and colleagues tested how both children and adults generalise a novel construction (in our terminology, equivalent to a rule). They find that people in both age groups are better at generalising this construction when they saw the construction applied to a skewed distribution of items.

The usage-based literature attributes this effect to the preponderance of one particular type, suggesting that it increases familiarity with the construction in a way that serves as a foundation for extension to new instances though analogy (e.g., Bybee, 2008). But another important property of skewed distributions is their long tail of low-frequency items. We’ll suggest that it’s this property, rather than the presence of one high-frequency type, that particularly aids rule generalisation.

### Other Distributional Accounts of Rule Productivity

Linguists specialising in corpus research have also proposed ways of quantifying a rule’s generalisability (which, in that tradition, is typically called productivity) based on distributional information. In this section we describe two widely-used measures commonly applied to distributional data: the Tolerance Principle (Yang, 2016) and potential productivity (Baayen, 2001). And in Section Existing Measures of Rule Productivity Do Not Match Our Experimental Results below, we apply them to our experimental data and show that neither quite fits the pattern we observed.

First, the Tolerance Principle is a simple mathematical expression that uses type counts to declare whether or not a rule *R* is productive (Yang, 2016). We first need to know *N*, how many types could potentially be used with *R*. From *N* we compute a threshold value *θ_N_*, how many types *e* are allowed to be exceptions to *R*. *θ_N_* is defined as

$$\theta_{N}=\frac{N}{\ln N}.$$

If *e* > *θ_N_*, then we declare the rule unproductive. Otherwise, if *e* ≤ *θ_N_*, we declare the rule productive.

The Tolerance Principle does not take token frequency into account at all. This could be what leads it to make some erroneous predictions about the outcome of our experiment (see Section The Tolerance Principle).

A conceptually very different measure is Baayen’s (2001, 2009) potential productivity *P*. For *P*, token frequency is central, in particular the number of types that appear with a token frequency of 1. These types are called hapax legomena.

Based on a sample of data derived using a given rule, *P* is defined as

$$P=\frac{\text{number}\;\text{of}\;\text{hapax}\;\text{legomena}\;\text{in}\;\text{the}\;\text{sample}}{\text{number}\;\text{of}\;\text{tokens}\;\text{in}\;\text{the}\;\text{sample}}.$$

One way to interpret *P* is as the probability of encountering a hapax legomenon in a sample of a given size.

As we’ll show below in Section Potential Productivity, the predictions *P* makes don’t align with our experimental results. But this is because the measure trades off comprehensiveness for greater ease of computation. It stops at hapax legomena and does not incorporate dis legomena (types with a frequency of 2), tris legomena (frequency 3), and so on, though we believe that these can also be crucial. Conceptually, though, we share Baayen’s thinking. What’s most important for a rule’s generalisability is how many low-frequency items (not just hapax legomena) it applies to.

### The Present Proposal

In the experimental literature mentioned above, linguists studied, largely independently, the effects of type count and skew on rule generalisation. In each of these two strands of research, the variable that’s not at issue is held constant: experiments on the effect of type count compare distributions over different numbers of types, but their shape is always uniform, and experiments on the effect of skew compare skewed and uniform distributions, but the type count of both is the same. Controlling these variables gives a tidy experimental design but limits the scope of possible explanations. The account we present here will explicitly take both dimensions into account.

The main contribution of this paper is to show, using a behavioural experiment in Section Rule Generalisation in an Artificial Language Learning Experiment and computational model in Section An Urn Model of Rule Generalisation, that both the facilitatory effect of type count and that of skew can be explained in terms of how many low-frequency items the distribution contains. This proposal connects to Baayen’s (2001) seminal work, where a central role is played by the items with the lowest frequency.

**Figure 1.**
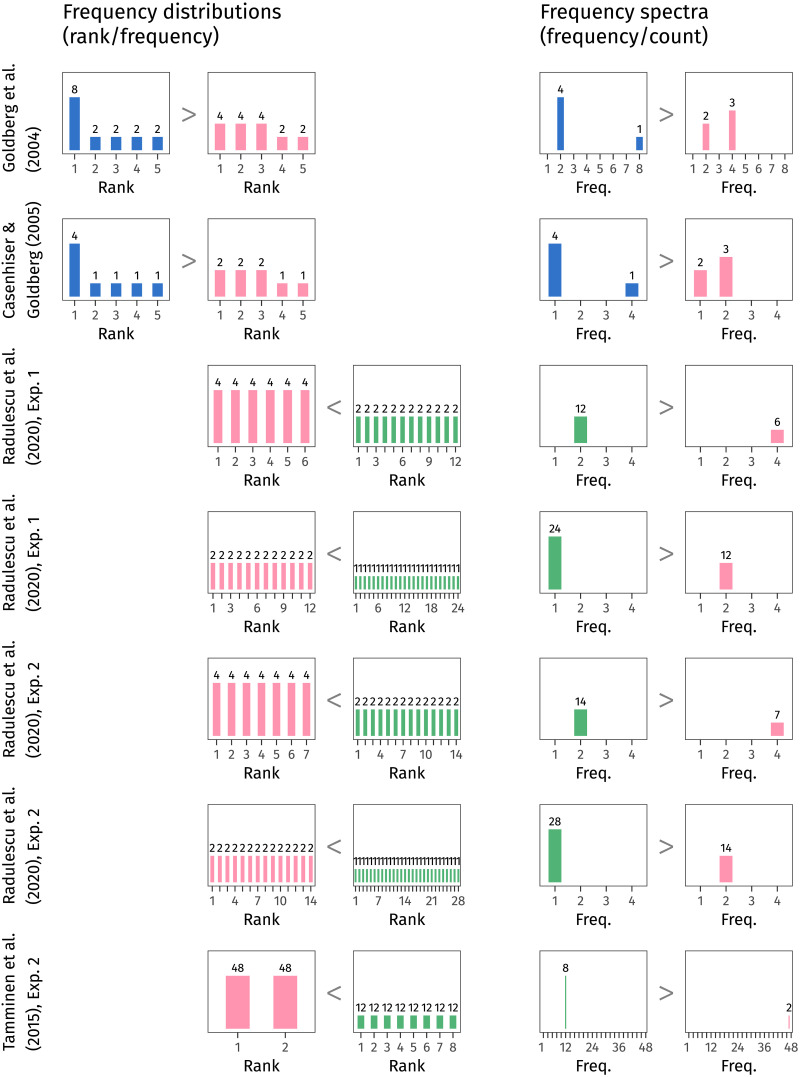
Frequency distributions and frequency spectra for the distributions tested in some previous studies on rule generalisation. The inequality signs reflect which distribution leads to better generalisation. When results are considered in terms of frequency spectra, which count up items with each frequency value, then previous findings can be unified in terms of an advantage for a greater number of low-frequency items.

In Figure 1, we illustrate what we mean by “how many low-frequency items the distribution contains”. The left section of the figure shows the frequency distributions that a selection of previous studies have tested. The inequality signs between them reflect which distribution leads to better rule generalisation. As discussed above, studies on skew (the top two rows) show an advantage for the skewed distributions; studies on type count (the bottom five rows) show an advantage for the distribution with more types.

It’s common practice to summarise the frequency of different types using a frequency distribution. It’s a bit less common to summarise the frequencies themselves, asking: how many times does this sample contain words with each frequency value? The answer to this question is given by a frequency spectrum (Baayen, 2001).

Like frequency distributions plot a word’s rank against its frequency, so do frequency spectra plot a frequency value against *its* frequency (which for clarity we call its count). Frequency spectra are useful because they illustrate clearly how many rare items appear in a sample. In the right section of Figure 1, we show the frequency spectra derived from each frequency distribution. This way of looking at previous experimental results suggests that, across the board, the distribution with more low-frequency types (that is, higher counts on the left edge of the frequency spectrum) leads to better generalisation.

We ran an artificial language learning experiment which replicates previous findings about the advantage of type count and skew for rule generalisation (Section Rule Generalisation in an Artificial Language Learning Experiment). Then, as a sense check, we implemented an urn model of learning which pits previous proposals from the literature against our own (Section An Urn Model of Rule Generalisation). We simulated learners that prefer to generalise a rule with a greater type count (as proposed by, e.g., Gómez, 2002), or relatedly following the Tolerance Principle of Yang (2016), or with a more skewed distribution (as proposed by, e.g., Casenhiser & Goldberg, 2005; Goldberg et al., 2004), or with more low-frequency items. Our experimental results were replicated only with the simulated learners that preferred to generalise the rule they saw with more low-frequency items.

We further suggest that, if language users do indeed interact with naturalistic language data in this way, their behaviour can be described in terms of rational Bayesian reasoning (Section General Discussion). We find results resembling the behaviour of our experimental participants from a Bayesian model that estimates how likely it is that the true population of types that a rule could apply to extends beyond the types that the learner has already seen.

The finding that when a rule applies to low-frequency words, it’s more likely to be generalised, suggests the insight that linguistic rule generalisation is a self-perpetuating process. This is because low-frequency words are the very ones that are *produced* when a language user applies a rule to a novel stem, and the newly-created word becomes a low-frequency item in somebody else’s input.

To briefly unpack the way language acquisition and language transmission feed into one another in this process: we find that one rule is more likely to be generalised if it appears with more low-frequency items than other rules do. And a generalised rule is the one that’s used (productively) for novel instances. So, a language user at time 0 (call them L0) who wants to produce some novel item will produce this novel item with the generalised rule.

Then a future language user at time 1 (call them L1) will encounter those items, formed using the rule that L0 had generalised. For L1, those items will likely be low-frequency, since they were just coined by L0. So, because generalisation tends to happen when rules are encountered with low-frequency items, L1 will generalise this same rule and use it productively for novel instances in turn.

In this way, rule generalisation is a self-reinforcing feedback loop: the more that people generalise with a particular rule, the more that other people will generalise with it too. And in this way, linguistic rule generalisation could be one process contributing to the presence of long-tailed distributions in language (Zipf, 1949); we return to this idea in Section On the Emergence of Skewed Distributions in Language.

## RULE GENERALISATION IN AN ARTIFICIAL LANGUAGE LEARNING EXPERIMENT

**Figure 2.**
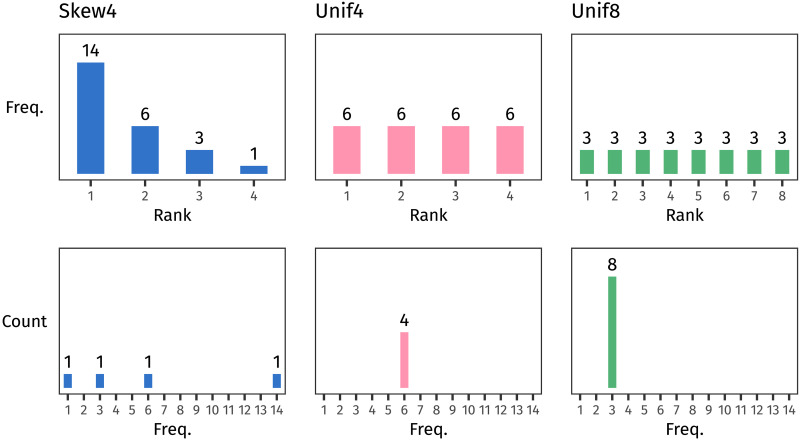
Frequency distributions (top row) and frequency spectra (bottom row) of the stems in each block of the experiment (three blocks in total). Participants in Group 1 learned two suffixes, one whose stems were distributed following Skew4 and the other with stems following Unif4. Participants in Group 2 also learned two suffixes, one following Unif4 and the other following Unif8. Because both Skew4 and Unif8 contain types with lower frequencies than Unif4, as the frequency spectra show, we would predict that both should prompt better rule generalisation.

As mentioned above, a number of experimental studies have used artificial language learning experiments to explore how distributional structure impacts generalisation. Here, we use this method to directly compare the potential facilitatory effects of two distributional features that have featured in previous literature: type count and skew. We did this by teaching participants an artificial language containing two plural suffixing rules and then testing them on which one they would apply to novel stems. In training, each of the two plural suffixes occurred with its own set of stems, and each set of stems followed its own frequency distribution (see Figure 2). One distribution, the baseline, has low skew and low type count (Unif4 in Figure 2). We manipulate between participants whether the other distribution has high skew (Skew4) or high type count (Unif8) relative to this baseline.

After training, participants were asked to choose which of the two suffixes to use with familiar stems as well as novel ones. Their choices on familiar stems let us verify that they learned the language they were trained on; their choices for novel stems let us investigate the effect of our manipulation on how they generalised each rule.

This experiment was preregistered with the Open Science Foundation.^1^ It was approved by the University of Edinburgh’s PPLS ethics committee (ref. 360-2223/2).

### Design

The stems followed one of three frequency distributions, visualised in Figure 2. The first is a skewed distribution of 24 tokens over four types; we call this distribution Skew4. The second distribution consists of 24 tokens distributed over four types uniformly. We call this Unif4. Finally, the third distribution is a uniform distribution of 24 tokens over eight types, called Unif8. We chose these distributions because the type frequencies in Unif4 and Unif8 both also appear in Skew4, allowing us to compare between them.

To compare all three distributions without requiring participants to learn three different plural rules, we divided participants randomly into two groups that would learn two rules each. Participants in Group 1 saw one plural rule apply to Skew4-distributed stems and another plural rule apply to Unif4-distributed stems; participants in Group 2 saw their two plural rules with stems following Unif4 and Unif8. Unif4 is thus the baseline distribution, encountered by all participants. Skew4 is the non-baseline distribution in Group 1, and Unif8 is the non-baseline distribution in Group 2. We included Unif4 as a baseline in both groups so that we could study the effect of a distribution’s shape in Group 1 and the effect of its type count in Group 2.

### Materials

The artificial language that participants learned consisted of plural forms of nouns, each composed of a stem and a suffix, e.g., *shoofmo* and *puthni*. The full set of stems is *bas, chul, cral, duk, fup, gloot, harg, jes, kuz, loor, puth, ruz, shoof, sleb, thap, tob, veb, wak, yeth*, and *zoof*. The two plural suffixes are -*mo* and -*ni*.

Every participant learned a slightly different language built out of these components randomly. For each participant, each suffix was randomly assigned to a distribution, and each stem was randomly assigned to a frequency rank. Stems were also mapped at random to digital illustrations of everyday items. Each illustration shows a pair of the following objects: a ball, a bell, a broom, a car, a clock, a curtain, a hammer, a mobile phone, a rocket, a ruler, a pair of scissors, and a toilet.^2^

### Procedure

The experiment was implemented using jsPsych (de Leeuw et al., 2023), and it ran in participants’ web browsers. Experiment code (as well as all data and analysis code) will be published with the Open Science Foundation.

At the beginning of the experiment, participants were told that they would learn a new language that has two different ways of saying that there is more than one object. Their task was to learn both ways. The experiment had three phases: the training phase, the practice phase, and the test phase.

**Figure 3.**
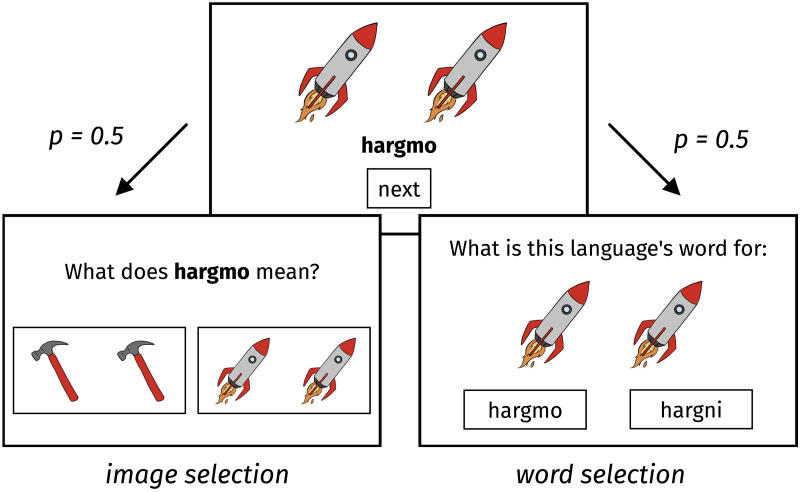
Each training trial consisted of a familiarisation trial (top), followed randomly by one of two types of practice trial (bottom). The practice trials were either an image selection task or a word selection task related to the image–word mapping from the familiarisation trial just before.

In the training phase, each trial began by familiarising participants with the object and the plural word (see Figure 3). The screen first displayed an image of a pair of objects (e.g., two rockets). Then after 1,000 ms, a plural word appeared below the image (e.g., *hargmo*). Then 1,000 ms after that, a “next” button appeared. Upon clicking “next”, participants saw a two-alternative forced choice (2AFC) practice trial that related to the image–word mapping in the familiarisation trial they had just seen. Participants either saw an image and had to select the correct word, or they saw a word and had to select the correct image. Each type of practice trial occurred with 50% probability. In the word selection trials, the foil word was the correct stem with the incorrect suffix. In the image selection trials, the foil image was drawn uniformly at random from the objects that appeared with the same suffix as the target (e.g., in Figure 3, the word for “hammers” would also use the suffix -*mo*). Participants saw feedback on their responses displayed on screen for 2,000 ms.

There were 48 training trials (that is, 48 pairs of familiarisation and practice trials), 24 per suffix, in each experiment block. The experiment contained three blocks, for 144 trials in total. The order of trials was randomised within each block for each participant.

Next, the practice phase consisted only of practice trials like the ones from the training phase: both word and image selection tasks, now with no preceding familiarisation trial, but still with feedback on participants’ responses. Every familiar image–word mapping appeared once as the target of a word selection task and once as the target of an image selection task. Consequently, the practice phase contained a different number of trials for each participant group. Participants in Group 1 learned eight types in total (four in Skew4, four in Unif4), so their practice phase was 16 trials long, while Group 2 learned twelve types (four in Unif4, eight in Unif8), so their practice phase was 24 trials long. (That Group 2’s practice phase was slightly longer does not seem to affect their accuracy on familiar items at test, as we will see in the overall learning accuracy results below.) The order of trials in the practice phase was fully randomised.

Finally, the test phase simply asked participants how someone who speaks this language would say there is more than one X. Participants were given two choices, Xni and Xmo, presented on buttons in a random order per participant. In eight of the sixteen test trials, X was a familiar stem seen during training, e.g., *harg*. (These eight trials tested four stems from each of the two distributions participants were trained on; for Unif8, four stems were sampled uniformly at random.) In the other eight test trials, X was a novel stem not encountered during training.

### Participants

We recruited 102 participants from Prolific’s pool of self-reported L1 English speakers resident in the United Kingdom who had previously participated in over 50 studies on Prolific and who had an acceptance rate of over 95%. Two participants reported in the debrief questionnaire that they took written notes to remember the language, counter to instructions, so following our preregistered exclusion criteria, we excluded their data from analysis. The experiment’s median completion time was around 22 minutes, and all participants were paid £4.15 for their time (above UK minimum wage at the time of running the experiment).

### Results

We focus first on how well participants learned the artificial language they were trained on before turning to the generalisation results.

#### Overall Learning Accuracy.

**Figure 4.**
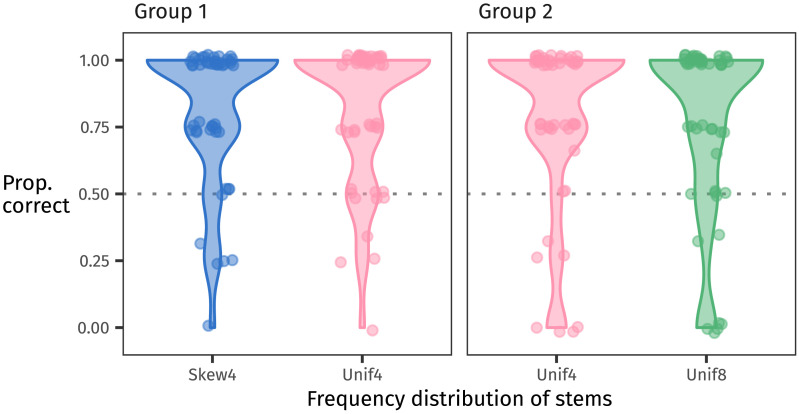
Proportion of correct responses at test to familiar stems for each group. Each dot represents one participant’s responses. The dotted line indicates chance at 50%, and overall accuracy well exceeds this level: 77 of 100 participants responded correctly to all familiar trials.

Accuracy on familiar items was high across the board, with 77 of 100 participants achieving 100% success (split 38–39 between groups; see Figure 4).

To estimate how learning accuracy might vary between different frequency distributions, we used brms in R (Bürkner, 2017; R Core Team, 2025) to fit a preregistered hierarchical Bayesian generalised linear model with a Bernoulli likelihood. The model’s weakly regularising priors were determined based on prior predictive checks (see Appendix A for the full model specification). The model estimated the log-odds of responding correctly as a function of the stems’ distribution, and it also included by-participant intercept adjustments. We coded the distribution variable using repeated contrasts (Nicenboim et al., 2024): the first contrast estimated the difference between Skew4 and Unif4, and the second contrast estimated the difference between Unif4 and Unif8. With this coding scheme, the intercept represents the grand mean: overall accuracy.

The model converged, as indicated by all Rhats = 1.00. The posterior probability distributions estimated for the population-level effects are summarised in Table 1. The large positive intercept reflects learners’ high accuracy; 1.80 log-odds (95% CrI: [1.46, 2.20]) is equivalent to a mean probability of responding correctly of 86% (95% CrI: [81%, 90%]). For both contrasts, both positive and negative values are considered plausible—these estimates indicate high uncertainty about any association between distribution and learning accuracy. Thus, all distributions appear to be learned to a similar, fairly high degree, a reassuring starting point for analysing how people generalise from a learned system.

**Table 1.** Summaries of the posterior probability distributions estimated for the learning data (values in log-odds space). The large positive intercept indicates reasonable certainty that accuracy is high, and the the other predictors indicate great uncertainty about any differences between distributions.

	Posterior mean	95% CrI (lower)	95% CrI (upper)
Intercept	1.80	1.46	2.20
Skew4 vs. Unif4	−0.06	−0.60	0.47
Unif4 vs. Unif8	−0.17	−0.69	0.35

#### Learning Accuracy by Word Frequency.

**Figure 5.**
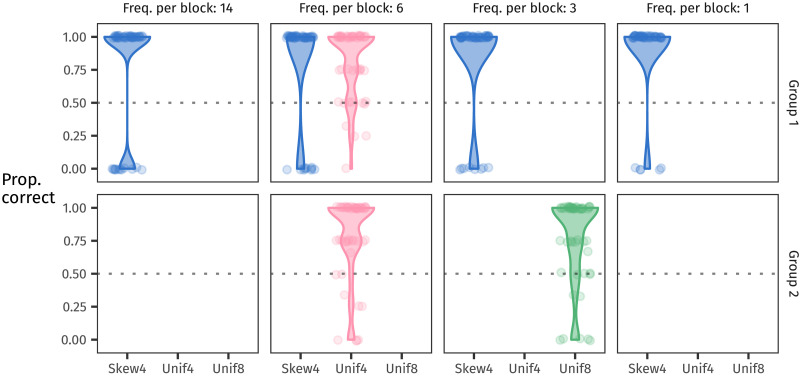
Participants’ accuracy on familiar words as a function of shown frequency, shown with decreasing frequency (i.e., increasing rank) from left to right. Each point represents a participant’s accuracy on words they saw a given number of times. For Skew4, participants only learned one word with each frequency value and were only tested on each word once, so their accuracy can only be 0 or 1. For Unif4 and Unif8, participants were tested on four words that share a frequency value, so there, the proportion of correct responses can fall in one-quarter increments between 0 and 1. Accuracy is generally high, but surprisingly, more participants respond correctly to low-frequency words than to high-frequency ones, at least for Skew4.

Intuitively, we might expect that participants would perform better on high-frequency words than low-frequency ones, and that when uniform and skewed distributions share a token count, words from a skewed distribution would be learned better (as found by, e.g., Hendrickson & Perfors, 2019; Lavi-Rotbain & Arnon, 2022; Wolters et al., 2024). We preregistered an exploratory analysis to investigate this question. Interestingly, Figure 5 shows the opposite pattern: accuracy increases as word frequency *decreases*, with no great difference in accuracy between the three distributions.

We fit a hierarchical Bayesian generalised linear model with a Bernoulli likelihood to this data (model specification in Appendix A). This model estimated whether the log-odds of choosing the correct suffix for a word are associated with that word’s frequency in the training phase (i.e., its per-block frequency times three). We logged and centered the word frequency variable, and included by-participant adjustments to the intercept and by-participant adjustments to the slope over logged centered word frequency.

The model converged (all Rhats = 1.00). Its posterior probability distributions for the population-level effects are summarised in Table 2. The Intercept estimate indicates that when logged word frequency is at its mean, the log-odds of choosing the correct suffix are 2.07 (95% CrI [1.64, 2.57]), equivalent to a probability of 89% (95% CrI [84%, 93%]). Increasing a word’s log frequency by one unit changes the log-odds of responding correctly by between −1.16 and 0.11 (with 95% probability). Thus the association between word frequency in training and correct responses is more likely to be negative—there is more posterior probability density allocated to negative values than positive ones—but the model still assigns some plausibility to a positive association too.

**Table 2.** Summaries of the posterior probability distributions estimated for the association between logged, centered word frequency and accuracy (values in log-odds space).

	Posterior mean	95% CrI (lower)	95% CrI (upper)
Intercept	2.07	1.64	2.57
Freq. (logged, centered)	−0.50	−1.16	0.11

If participants learned high-frequency words more reliably than low-frequency ones, then we would expect this association to be positive. That the model considers a negative association so plausible is at first glance counter-intuitive, but we’ll discuss a possible explanation of this result in Section Interim Discussion below. First, though, we turn to the main goal of the experiment: the generalisation results.

#### Generalisation.

In this section, we describe two different analyses of the generalisation data. First we analyse the data from all 100 participants, following our preregistered analysis plan. Then we analyse only the data from the 77 participants who learned the language perfectly, that is, who responded to all familiar words with 100% accuracy.^3^

##### Analysis of All Participants.

**Figure 6.**
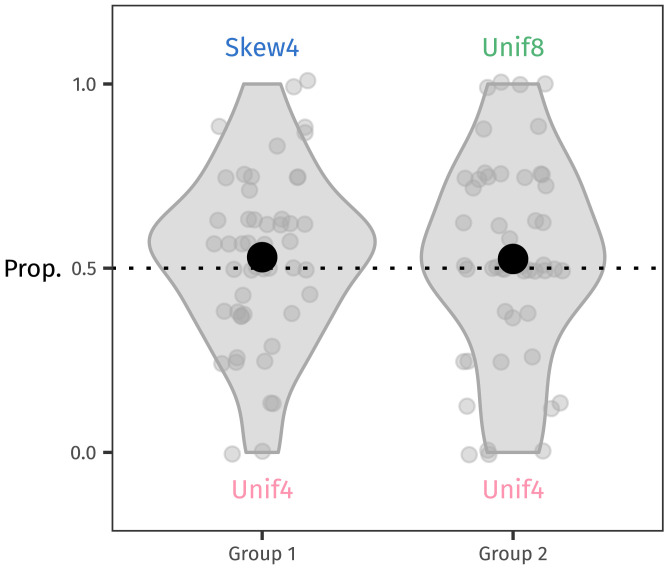
For all 100 participants, the proportion of non-baseline suffixes chosen for novel stems at test. Each point represents one participant’s responses. The dotted line at 50% reflects where responses would fall if they were probability matching. Many participants cluster around this level; no clear preference for the non-baseline suffix is evident.

The proportion of suffixes that each participant chose for the eight novel stems they saw is shown in Figure 6. In line with our predictions, numerically slightly more participants selected the non-baseline suffix than the baseline one in each group. However, many participants clustered around 50%. Participants’ input did contain each suffix 50% of the time, so replicating that balance by “probability matching” (Gaissmaier & Schooler, 2008; Hudson Kam & Newport, 2005) is a reasonable approach.

The aim of this part of the study was to see whether participants in each group preferred to generalise with their respective non-baseline suffix, and whether one distributional property or the other—skew or increased type count—was more likely to prompt generalisation. So, following our preregistered analysis plan, we fit a hierarchical Bayesian generalised linear model with a Bernoulli likelihood to the generalisation data. This model estimated the log-odds of selecting the non-baseline suffix as a function of participant group, again including by-participant intercept adjustments. The group predictor was sum-coded with Group 1 (effect of skew) as −0.5 and Group 2 (effect of type count) as +0.5. The intercept thus represents the grand mean, estimating how likely participants were overall to generalise with their non-baseline suffix.

The model converged (all Rhats = 1.00). Posterior probability distributions for the population-level parameters are summarised in Table 3. The Intercept estimate indicates that, while participants might lean toward the non-baseline suffix (since the model allocates slightly more posterior probability density to positive values), the observed data might also be consistent with the opposite tendency, or no preference at all (i.e., 50% probability matching or responding at chance). The model’s estimates also reflect great uncertainty about how the preference strengths in each group might differ.

**Table 3.** Summaries of the posterior probability distributions estimated for the generalisation data for all 100 participants (values in log-odds space). These estimates suggest uncertainty about any overall preference for the non-baseline suffix, and there is particularly great uncertainty about any difference in this preference between groups.

	Posterior mean	95% CrI (lower)	95% CrI (upper)
Intercept	0.13	−0.12	0.37
Diff. between groups	−0.02	−0.49	0.46

##### Analysis of Perfect Learners.

**Figure 7.**
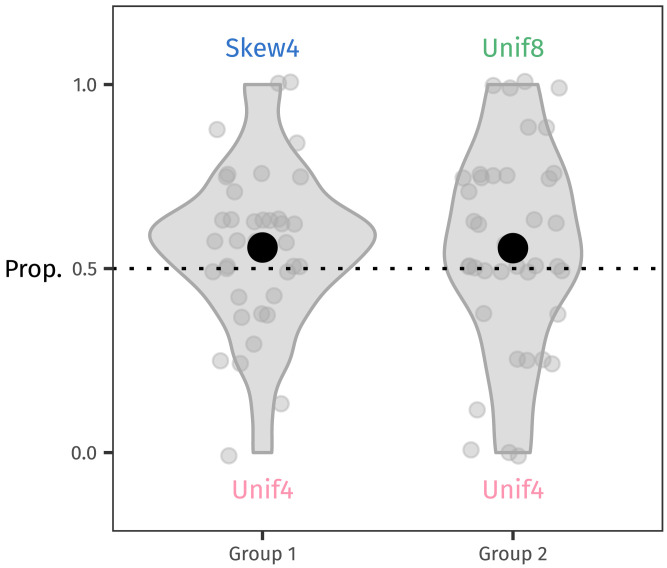
Each participant’s proportion of non-baseline suffixes chosen for novel stems at test, shown for perfect learners only (*N* = 77). The dotted line at 50% reflects where participants’ responses would fall if they were probability matching or answering at chance. The tendency to generalise using the non-baseline suffixes is more pronounced among the perfect learners.

This analysis is exploratory and was not preregistered; see Footnote 3. Of the 77 perfect learners, 38 were in Group 1 and 39 were in Group 2. The generalisation behaviour of this subset of participants is visualised in Figure 7. We fit the same model as in the previous section to this data, and Table 4 summarises its posteriors.

**Table 4.** Summaries of the posterior probability distributions for the generalisation data for perfect learners only (values in log-odds space). The intercept (grand mean) is almost entirely above zero, suggesting that perfect learners are much more likely to select the non-baseline suffix than the baseline suffix, though a very little uncertainty remains. This model remains highly uncertain about any difference between groups.

	Posterior mean	95% CrI (lower)	95% CrI (upper)
Intercept	0.26	−0.004	0.53
Diff. between groups	0.01	−0.53	0.55

This time, the 95% CrI of the intercept is almost entirely positive, suggesting that perfect learners are almost certainly more likely to generalise using their respective non-baseline suffix: in Group 1, this is the suffix learned with Skew4, and in Group 2, the suffix learned with Unif8. Further, the model’s estimates still reflect great uncertainty about any differences between how much each group prefers their non-baseline suffix.

### Interim Discussion

In this artificial language learning experiment, we sought to replicate previous findings about what properties of frequency distributions facilitate rule generalisation: a greater type count, on the one hand, and a skewed shape, on the other. We taught participants two plural rules and manipulated the distribution of stems that each rule applied to. Then we asked which rule they would prefer to generalise to brand new stems.

The baseline frequency distribution was a uniform distribution over four types, Unif4. In Group 1, we pitted Unif4 against a skewed distribution over four types, Skew4. Participants in Group 1 who learned the language perfectly did indeed show a small preference to generalise with the rule they saw with Skew4. And in Group 2, we pitted Unif4 against another uniform distribution, but one that has a greater type count: Unif8. Again, perfect learners showed a small preference to generalise with the rule they saw with Unif8. Our results thus replicate both the advantage for skew and for a greater type count.

As mentioned above, previous work on the effect of type count (Gómez, 2002) explained the advantage for a greater type count in terms of variability, and this idea also connects to our results here. Greater variability in the stems can help the invariant parts of the context, the suffixes, stand out. And low-frequency types contribute to greater variability.

However, our results also revealed a surprise: participants did not show the expected positive association between word frequency in training and accuracy at test. Rather, participants tended to respond more accurately to items that they saw *less* often, in particular when responding to items drawn from the skewed frequency distribution. Even though the Bayesian model indicates some uncertainty in the direction of this effect, that it is not clearly positive is striking. In previous word learning studies like Hendrickson and Perfors (2019), Smith et al. (2023), and Wolters et al. (2024), high-frequency words are learned better than low-frequency ones.

We suggest that this result could have emerged because, although these trials were intended to test learning, participants may already have been generalising. In particular, participants might have robustly learned one set of stem–suffix mappings and relegated any stems which they did not recognise as part of that set to the other suffix.

To illustrate: participants might not have memorised all the -*ni* stems *and* all the -*mo* stems—instead, they might have memorised the set of -*ni* stems, say, and whenever they saw a stem that they didn’t recognise from the -*ni* set, they selected -*mo*. In other words, they may have treated -*ni* suffixation as lexically conditioned and taken -*mo* as the generalised, default rule.^4^

**Figure 8.**
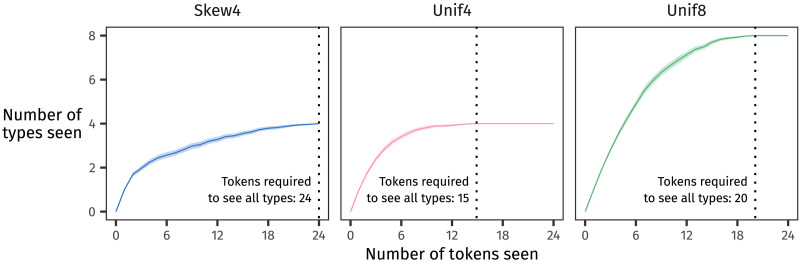
Vocabulary growth curves show how many types have been encountered in a sample of *n* tokens. These curves illustrate the rate at which participants would encounter types from each distribution during one block of the experiment; at the end of one block, all types will have been observed. The solid line represents the mean number of types for each token count, averaged over 100 randomisations of trials in the training phase, and the shaded ribbon around the mean shows the 95% confidence interval. Vertical dotted lines in each panel indicate the maximum number of tokens that need to be seen before all types have been encountered; fewer tokens are needed for Unif4 than for the other two distributions.

Why should it be -*mo* that’s treated as the general rule in this case? Because, likely, -*mo* is the one that appeared with the longer-tailed distribution. The more a suffix applies to infrequent stems, the harder it would be to memorise all those stem–suffix mappings^5^—and additionally, the longer it would take. Figure 8 illustrates that participants are, on average, more likely to encounter all four types from Unif4 before they encounter all types from Skew4 or Unif8. Participants would thus have an easier time memorising Unif4, and so they would be more likely to apply the suffix seen with Skew4 and Unif8—the long-tailed distributions—to low-frequency and novel stems. In our present example, that suffix is -*mo*.

So, to summarise: The co-occurrence of low-frequency items with -*mo* in participants’ input makes participants more likely to generalise the -*mo* pluralisation rule. But participants may not remember the identity of those low-frequency items. So participants generalise, applying -*mo* not only to the novel types they’ve never seen before but also to the low-frequency types that they may not have represented as strongly in memory. And this behaviour masquerades as high accuracy on our 2AFC test of learning.

To determine whether this explanation is on the right track, we could envision including a certainty rating task after training, asking participants how confident they are that they’ve seen a given stem before. Then we can see whether their ratings vary by frequency, and in particular whether low-frequency stems are rated similarly to entirely novel ones. If they are, this result would support our suggestion that participants are essentially treating low-frequency stems and novel stems the same, generalising some default suffix to both.

### Formalising Our Proposal

As mentioned above, accounts of rule generalisation previously proposed in the literature have tended to focus on one distributional property or the other: either type count or skew. Type-count-based explanations point to the variability introduced by a large number of distinct items (e.g., Gómez, 2002; Radulescu et al., 2020)—but if this is all that’s going on, then the difference between uniform and skewed shapes, when type count remains constant, remains unexplained. And skew-based explanations consider the one high-frequency type to play a crucial role (e.g., Casenhiser & Goldberg, 2005; Goldberg et al., 2004), but this doesn’t predict an advantage for a greater number of types. But as we’ve suggested here, both accounts can likely be unified by an explanation in terms of which distribution contains more rare items.

So far, though, we’ve just been dealing in intuitions. In the next section, we move beyond narrative argumentation to formalise these explanations using a model of learning and generalisation. We will compare our proposal to other explanations from the literature by simulating what our experimental results would look like under each account, and we will show that only the low-frequency preference accords with empirical results.

## AN URN MODEL OF RULE GENERALISATION

The kind of model we adopt here is known as an “urn model” (Keogh et al., 2024; Keogh & Pankratz, 2024; Spike et al., 2017). Traditionally, urn models represent learners as a collection of mappings between meanings and signals. For every meaning, a simulated learner has an “urn”. Every time the learner receives a signal associated with a given meaning, one token of that signal is placed in that meaning’s urn. And when a learner is prompted to convey a particular meaning, it stochastically chooses a token from that urn.

We adapt the traditional urn model slightly here: in our version, each urn represents not one semantic category but one distributional category. Each learner has one urn per rule (one for -*mo* and one for -*ni*), and each urn accumulates all the forms that the learner sees with each rule. We define the generalisation task in this urn model framework as the decision of which urn to add a novel stem to.

Our generalisation task resembles a Chinese restaurant process, a stochastic process which has been widely used in modelling language and cognition because it partitions items into categories, the sizes of which follow a power law (see, e.g., Goldwater, 2007; Goldwater et al., 2009; Pearl & Phillips, 2018; Tenenbaum et al., 2011). The basic intuition of the Chinese restaurant process is that the rich get richer: items are added to existing categories with a probability proportional to the number of items already in that category.

The “Chinese restaurant” metaphor frames this process in terms of diners seating themselves at restaurant tables. Diners are also permitted to choose an unoccupied table in classic versions of the Chinese restaurant process, but we restrict our simulated learners to only two tables, that is, two urns: -*mo* and -*ni*.

By selecting each urn with a probability proportional to how many tokens it contains, our model captures the probability matching strategy for free. And by storing individual tokens, each urn’s contents are easy to summarise as a frequency distribution—important for computing properties of this distribution that might cause the simulated learner to prefer one urn over another. These distributional properties weight the outcome of the stochastic process in favour of different urns, depending on which distributional properties the learner prefers. We refer to these preferences for different properties as “generalisation strategies”.

### Six Different Generalisation Strategies

Based on proposals from the literature we have been building on (e.g., Casenhiser & Goldberg, 2005; Goldberg et al., 2004; Gómez, 2002; Radulescu et al., 2020; Tamminen et al., 2015; Yang, 2016), we implemented six different strategies that simulated learners might use for deciding which urn to generalise with.

First, to serve as a null condition, we implemented pure probability matching. Simulated learners who probability match are not swayed by any properties of their urns’ frequency distributions. They simply select each urn with a probability proportional to the number of tokens each urn contains. (This version of the urn model is most akin to the classic Chinese restaurant process described above: novel types are allocated to urns purely based on how many tokens the urn already contains.)

Next, we drew on the research suggesting that people generalise the rule they see with a greater number of types (e.g., Gómez, 2002; Hopman, 2022; Radulescu et al., 2020; Tamminen et al., 2015; Valian & Coulson, 1988). Simulated learners with this strategy prefer to select the urn with the largest type count.

As a variant of the type count approach, we implemented a strategy based on the Tolerance Principle of Yang (2016). The Tolerance Principle offers a slightly more nuanced take on how type counts contribute to a rule’s productivity; see Section Other Distributional Accounts of Rule Productivity above. Simulated learners with this strategy prefer to select the urn that the Tolerance Principle considers productive. (We do not include a strategy based on Baayen’s potential productivity measure for reasons that will become clear in Section Existing Measures of Rule Productivity Do Not Match Our Experimental Results below—in short, potential productivity would yield no preference for either urn.)

We implemented the preference for a skewed distribution (Casenhiser & Goldberg, 2005; Goldberg et al., 2004) in two different ways. Goldberg and colleagues attribute the generalisation effect to the presence of one high-frequency type, so the first group of skew-preferring simulated learners tend to select the urn whose Rank 1 type has the highest frequency (regardless of the frequencies of Ranks ≥2). Another way to measure skew is to summarise the entire distribution using Shannon entropy (see, e.g., Lavi-Rotbain & Arnon, 2021, 2022; Shufaniya & Arnon, 2022). The Shannon entropy of a probability distribution over a random variable *X* is defined as

$$H\left(X\right)=-\sum_{x\in X}p\left(x\right)\log_{2}p\left(x\right).$$

Entropy is maximised when a distribution is uniform, and it decreases as a distribution becomes more skewed. So the second group of skew-preferring simulated learners tend to select the urn whose frequency distribution has the lowest entropy.

The final strategy encodes the explanation we have offered here, inspired by work by Baayen (2001): a preference for the urn that contains a greater number of low-frequency types. Simulated learners with this strategy compare the frequency spectra in each of their urns, beginning with the lowest frequency. First, which distribution has more types with a frequency of 1 (that is, hapax legomena)? If this count is tied, which distribution has more types with a frequency of 2? And so on. Learners continue comparing their urns in this manner until one contains a larger number of low-frequency types than the other—this is the urn that these learners prefer.

For all these criteria, in the case of a tie between urns, the preferred urn is sampled uniformly at random.

Note that the urn that is preferred based on these criteria is not necessarily the one that the simulated learners will choose to generalise with. Always selecting the preferred urn would not be faithful to the experimental results, because it doesn’t take participants’ probability matching into account. Instead of deterministically choosing the preferred urn, learners are probabilistic: they begin with a probability matching strategy by considering the relative token counts in each urn, and then they modify these counts by up-weighting the preferred urn. We expand on this process below.

### Simulating Generalisation

**Figure 9.**
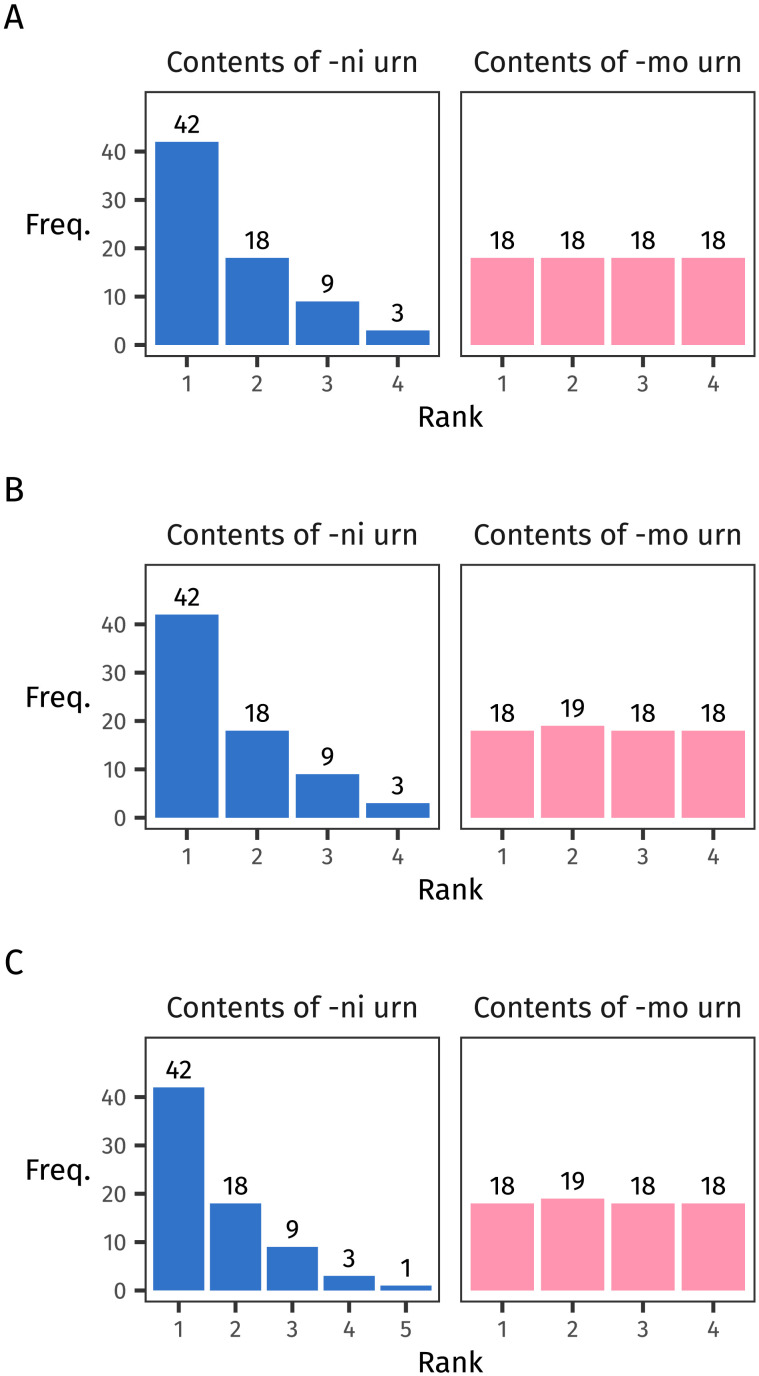
(A) After training, a Group 1 learner’s urns contain the same distribution of tokens that a participant saw in the training phase of the experiment. (B) In the test phase, a familiar token (here the Rank 2 type for -*mo*) is added to the urn, increasing that type’s frequency. (C) A novel token is added to the chosen urn (here introducing a type at Rank 5 in the -*ni* urn).

As in our experiment, half of the simulated learners were allocated to Group 1 (Skew4 vs. Unif4), and the other half to Group 2 (Unif4 vs. Unif8). Each learner received the same amount of input as in the experiment’s training phase, a total of 144 tokens. In each learner’s internal representation were two urns, one per suffix, and after training, each urn contained 72 tokens. Panel A in Figure 9 represents a Group 1 learner’s urns at the end of the training phase.

After training, we simulated the test phase by showing simulated learners eight familiar stems (four from each suffix) and eight novel stems in a randomised order. Learners who see a familiar stem respond with the correct urn (since we are simulating the behaviour of the perfect, error-free learners). They also add a token of this stem to the correct urn (Panel B of Figure 9).

Learners who encounter a novel stem must generalise, or in urn model terms, they must select which urn should gain the novel stem. Their selection is informed by whichever distributional property of their urns they care about, but as mentioned above, they do not deterministically generalise with the urn they prefer. Instead, they count the tokens in each urn, and then augment the preferred urn’s token counts by some value. These augmented counts are normalised to become a probability distribution over urns, and learners use this distribution to probabilistically select one urn or the other. How much a learner augments its urn by is sampled for each learner from a Poisson distribution. This amount represents the learner’s preference strength. The Poisson distribution’s *λ* parameter gives the mean preference strength and is fixed for each run of the simulation.

In more formal terms, where *n* represents the number of tokens in an urn:

$$k∼\text{Poisson}\left(\lambda \right)$$

$$u_{\text{pref}}=n_{\text{pref}}+k$$

$$u_{\text{dispref}}=n_{\text{dispref}}$$

$$U=\left[\frac{u_{\text{pref}}}{u_{\text{pref}}+u_{\text{dispref}}},\frac{u_{\text{dispref}}}{u_{\text{pref}}+u_{\text{dispref}}}\right]$$

and the chosen urn is sampled with the respective probability from *U*. (In principle, this process doesn’t need to be limited to two urns, but for simplicity we represent the two-urn version here.)

To illustrate: let some learner *A*’s generalisation strategy be a preference for low entropy. *A*’s internal representation is the one in Panel B of Figure 9. *A* computes the Shannon entropy of each urn: 1.5 bits for the -*ni* urn and just under 2 bits for the updated -*mo* urn. Because the Shannon entropy of the -*ni* urn is lower, -*ni* becomes *A*’s preferred urn.

Now let *A*’s preference strength be 45 (plausibly sampled from Poisson(50), one of the *λ* values we use in our simulation below). The token count in the -*ni* urn, 72, is augmented by 45 to become 117, while the token count in the -*mo* urn remains 73. Normalising these counts results in about a 62% probability of generalising with -*ni* and a 38% probability of generalising with -*mo*.

Again, in more formal terms:

$$45∼\text{Poisson}\left(50\right)$$

$$u_{ni}=72+45=117$$

$$u_{mo}=73$$

$$U=\left[\frac{117}{190},\frac{73}{190}\right]=\left[.62,.38\right]$$

In this instance, imagine that *A* chooses -*ni*. *A* would add the novel stem to the -*ni* urn, updating the distribution as shown in Panel C of Figure 9.

### Results

**Figure 10.**
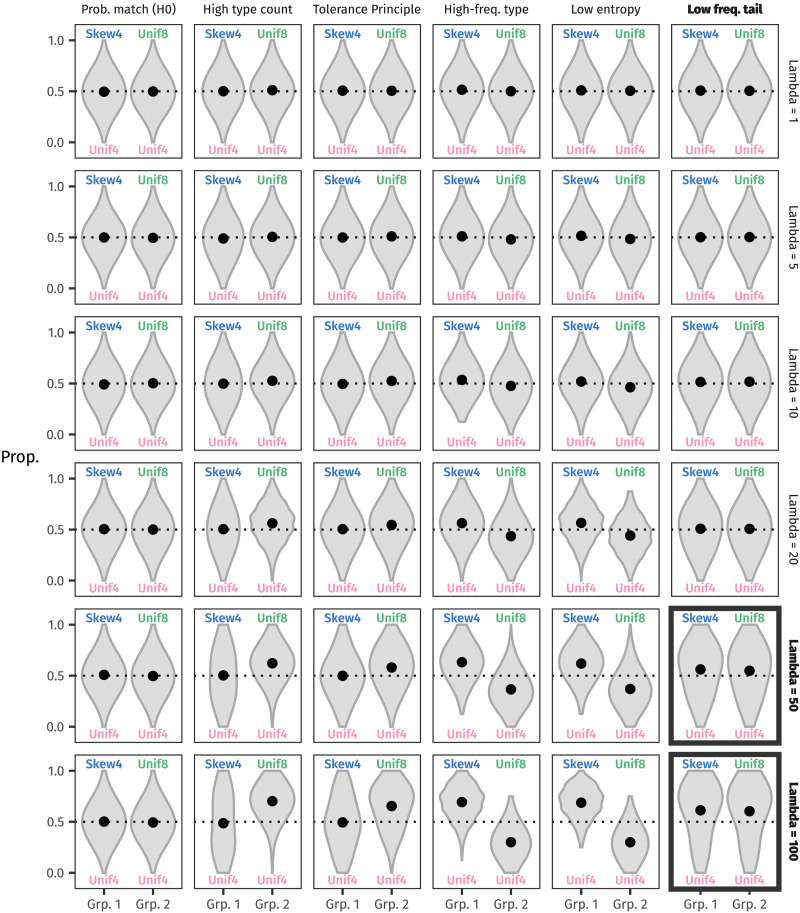
The proportion of non-baseline urns chosen by each simulated learner, for all combinations of generalisation strategies and preference strengths *λ*. The black circles represent the mean of each distribution of responses. Only the low-frequency tail strategy with a preference strength on a similar scale to the number of observed tokens (*n* = 72) reproduces the behaviour of participants in our experiment: Skew4 and Unif8 are both preferred equally over Unif4. The bolded boxes at bottom right show this result most clearly.

For every combination of generalisation strategy and preference strength *λ* ∈ {1, 5, 10, 20, 50, 100}, we computed how 1,000 simulated learners would respond to a simulated test phase. Figure 10 shows the results for the novel stems (analogous to Figures 6 and 7 above).

Reassuringly for a null condition, the mean of the probability matching strategy always remains at 50%. Both urns contain the same number of tokens after the simulated training phase, so each urn is always equally likely to be generalised with. But as *λ* increases, each of the other generalisation strategies shows its hand more and more.

When simulated learners prefer to generalise using the urn with a higher type count, Unif8 tends to win out over Unif4, since Unif8 starts out with more types than Unif4. And as it gains more novel types, it remains more likely to be chosen. In contrast, Skew4 and Unif4 stay balanced in the mean, because their type counts are equal.

A similar pattern emerges for the Tolerance Principle strategy. The Tolerance Principle considers the Unif8 rule to be productive when the Unif4 types are taken as exceptions (see the working-out below in Section Existing Measures of Rule Productivity Do Not Match Our Experimental Results). Thus, simulated learners with this strategy prefer to generalise with Unif8 over Unif4. However, neither Skew4 nor Unif4 are productive when the other distribution’s types are taken as exceptions (again, see Section Existing Measures of Rule Productivity Do Not Match Our Experimental Results), so simulated learners show no preference for either one.

Interestingly, both ways of measuring skew—as a preference for a higher-frequency Rank 1 type and as low Shannon entropy—yield equivalent results: Skew4 wins out over Unif4, and Unif4 wins out over Unif8. In terms of high-frequency types, Skew4’s Rank 1 type has a frequency of 14 after training, which beats Unif4’s Rank 1 frequency of 6; and Unif4’s Rank 1 frequency of 6 beats Unif8’s Rank 1 frequency of 3. And in terms of Shannon entropy, a skewed distribution has lower entropy than a uniform distribution over the same number of types (Skew4’s entropy is 1.52 bits, compared to Unif4’s 2 bits). And a uniform distribution over fewer types has lower entropy than a uniform distribution over more types (Unif4’s 2 bits wins out over Unif8’s 3 bits).

Crucially, only the preference for a greater number of low-frequency items captures the behaviour of participants in our experiment: both Skew4 and Unif8 are preferred to a similar degree over Unif4. At first, immediately after training, Skew4 and Unif8 both contain rarer types than Unif4—Skew4’s rarest type has a frequency of 1 and Unif8’s has a frequency of 3, compared to Unif4’s 6. And as novel types are added to the chosen urn, that urn’s frequency distribution accumulates ever more types with a frequency of 1, reinforcing the simulated learners’ preference.

We take these convergent results between experiment and model as confirmation that it is the observation of low-frequency types which prompts learners to generalise. But *why*? In the next section, we discuss one possible answer.

## GENERALISATION AS RATIONAL BAYESIAN INFERENCE

In this section, we will summarise a model showing how participants’ behaviour in the generalisation task can be captured by thinking of participants as rational Bayesian agents who reason about how likely it is that they have not yet encountered all the words that a rule could apply to. More concretely, the goal of this model is to simulate a participant’s reasoning in a single test trial of the experiment. After encountering training data that contains two different frequency distributions, what happens when a participant encounters a single novel stem and is forced to choose which rule to use?

The intuition behind the model is this: If you think you’ve already seen all the words that could be pluralised with a particular rule, then when faced with a new word to pluralise, you’re not likely to draw on that rule. But if you guess that there probably *are* other words out there that this rule could apply to, then when you encounter a novel word, the rule becomes a more viable option. And your reasoning about whether or not the rule could apply to types beyond the ones you’ve seen can be affected, we claim, by the distributional properties of the sample you’ve received. In particular, samples with a greater quantity of low-frequency items (Skew4 in the comparison of Skew4 vs. Unif4, and Unif8 for Unif4 vs. Unif8) are likely to come from latent populations that contain low-frequency, low-probability types.

**Figure 11.**
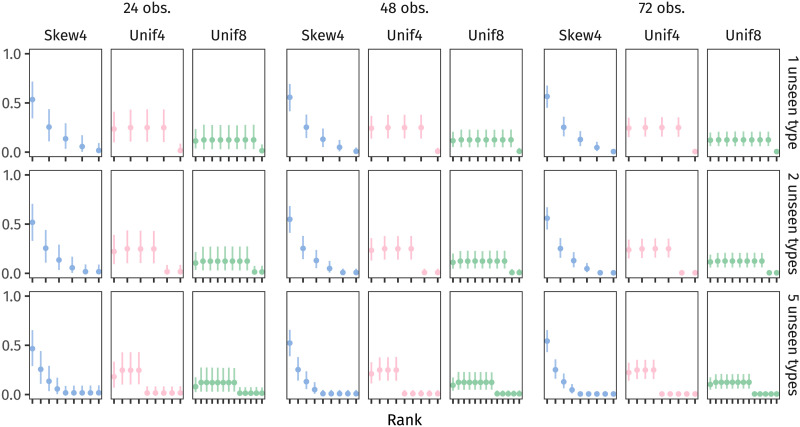
The conditional posterior probability distributions—that is, the plausible probabilities for each word type, both seen and unseen—estimated from samples of observed data. Points represent posterior means, and the horizontal lines span each type’s 95% CrI.

The first step of our model is to estimate a plausible latent population distribution based on the observed frequency distribution of stems. We used brms to estimate for each observed distribution Skew4, Unif4, and Unif8 a corresponding categorical distribution over the observed number of types (4, 4, and 8, respectively) + *n* unseen types where *n* ∈ {1, 2, 5}. We included these counts *n* to understand how people’s estimates may change, depending on how much bigger they imagine the latent population to be. Also, to see the effect of different amounts of input data, we considered samples containing 24 tokens, 48 tokens, and 72 tokens (in the experiment, participants observed 72). The estimated population distributions, using brms’s default priors, are shown in Figure 11. (All of this model’s code will be made available on OSF.)

Figure 11 shows that, as the number of observations increases (moving from left to right), the model’s uncertainty about each type’s probability decreases: the 95% CrIs of each type’s conditional posterior distribution become narrower. This makes sense—as the model gains information, it becomes more confident in its estimates.

Additionally, though, as observations increase, the model allocates less and less probability to the unseen types. This reflects how unlikely it is that such large samples would have failed to observe these additional words.

Further, as the number of unseen types in the latent population increases (in the three rows from top to bottom), the model reallocates some of the observed types’ probability to the unobserved types.

With these estimated latent populations in hand, the next step is to figure out how likely it is to draw a sample from each population that fails to observe every type—just like the training data failed to observe every type. To do this, we took advantage of Bayesian models’ generative capacity, generating 8,000 datasets from each latent population’s posterior predictive distribution. These datasets reflect what plausible samples generated by the population could look like (see Nicenboim et al., 2024, Section 3.6 for more detail on the posterior predictive distribution).

**Figure 12.**
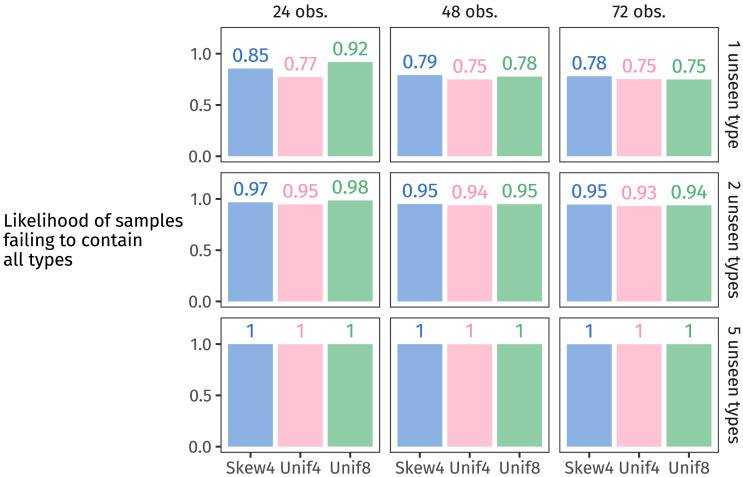
The proportion of datasets generated from each posterior predictive distribution that fail to contain all types in the population.

We then computed the proportion of those 8,000 datasets which failed to contain every one of the latent population’s types. This proportion approximates the likelihood we’re after. The likelihood for each estimated population distribution is shown in Figure 12.

In almost all cases, Unif4’s likelihood is lower than the other two distributions: Unif4 is less likely to yield missing types, compared to Skew4 and Unif8. Or in other words, observing all of the types in Unif4’s population distribution is more likely than for the others.

As samples get bigger (from left to right in Figure 12), the probability of missing types becomes lower—or phrased the other way around, larger samples are more likely to cover the whole population. However, as the assumed population gets larger (from top to bottom), observing all unseen types becomes increasingly unlikely. In other words, those unseen types are each so low-probability that failing to observe them all—especially when there are five—becomes near inevitable.

Now we have the likelihood of failing to observe all types, given a distribution. But what we want is the reverse: the probability of a distribution, given that we have failed to observe all types. In other words, we want the posterior probabilities of the frequency distributions that appeared in each participant’s input.

**Figure 13.**
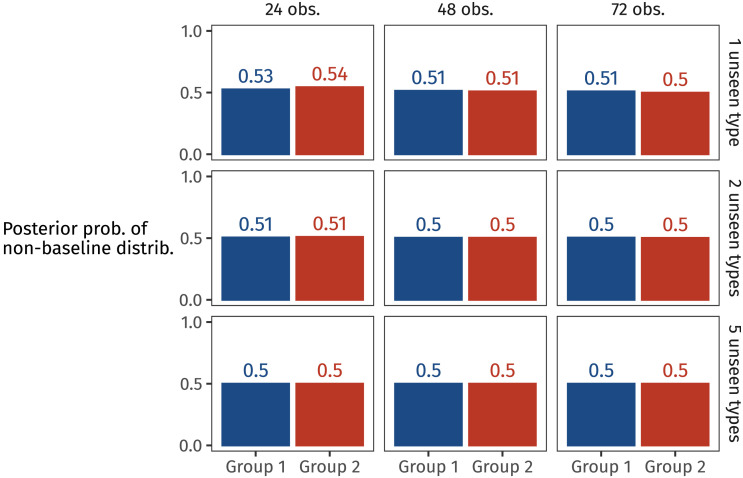
The posterior probability of each group’s non-baseline distribution (Skew4 for Group 1, Unif8 for Group 2) for a range of different observed token counts and posited number of unseen types in the population.

To arrive at these posterior probabilities, we can use Bayes’ Law. We assume uniform priors, since during training, participants saw each distribution an equal number of times. This assumption lets us simply normalise the likelihoods for each participant group’s hypothesis space (Skew4 and Unif4 for Group 1, Unif4 and Unif8 for Group 2). Figure 13 highlights the posterior probabilities of each group’s non-baseline distribution.

We see in Figure 13 that as the number of observed tokens increases (from left to right), and also as the number of unseen types increases (from top to bottom), the preference for generalising with the non-baseline suffix disappears and both suffixes become equally likely. The results largely align with participants’ generalisation behaviour, under certain assumptions. Assuming that participants reason one novel type at a time, rather than imagining that the population is far larger than the sample they encountered, then the non-baseline suffix receives slightly more posterior probability—as long as a small enough amount of data has been observed. As the number of observations increases, learners have ever less cause to believe that there are types beyond the ones they’ve seen. So they generalise with one affix over the other less and less (consistent with the rational behaviour observed in, e.g., Experiment 4 by Reeder et al., 2013). Participants in our experiment saw 72 tokens, by which point this model suggests that participants should be around chance, preferring a probability matching approach, which indeed they are.

A testable prediction resulting from this model is that, if learners are behaving rationally, then their generalisation behaviour should become more polarised toward the non-baseline distribution—the one with a longer tail—the fewer overall tokens they have observed.

## GENERAL DISCUSSION

In previous work on what properties of frequency distributions facilitate rule generalisation, linguists have focused (largely independently) on the effects of type count and of skew (for type count, e.g., Gómez, 2002; Tamminen et al., 2015; Valian & Coulson, 1988; for skew, e.g., Casenhiser & Goldberg, 2005; Goldberg et al., 2004). But because these properties have so far been considered separately, the explanations proposed fail to encompass them both. In particular, an explanation based solely on type count fails to predict that a skewed distribution should prompt generalisation better than a uniform one does. And an explanation based solely on skew fails to predict that, when two uniform distributions are pitted against one another, the distribution with more types wins out.

Here, we have unified these previous findings into a single account of linguistic rule generalisation, supported by an artificial language learning experiment and an urn-based learning model: people tend to generalise those rules to novel items that they’ve seen applied to more low-frequency types. This explanation may account for the type count advantage observed in many experiments, in which type count is manipulated but the number of tokens is held constant. A distribution with more types therefore has fewer tokens per type—each type becomes overall less frequent. In addition, this explanation may also account for the advantage conferred by skewed frequency distributions even when type count is held constant: skewed distributions have a long tail of low-frequency types.

Our preregistered artificial language learning experiment (Section Rule Generalisation in an Artificial Language Learning Experiment) supported this intuition by replicating both previous findings in a single design, showing that both skew and a greater type count tend to facilitate rule generalisation to a similar extent. Then an urn model (Section An Urn Model of Rule Generalisation) simulated how participants would behave in the experiment under different explanations of linguistic rule generalisation drawn from the literature. Some simulated learners preferred to generalise the rule that they saw with a greater type count, others preferred skew, and others still preferred a large number of low-frequency items. Only simulated learners who generalised based on low-frequency items reproduced the behaviour of participants in our experiment.

### Existing Measures of Rule Productivity Do Not Match Our Experimental Results

In this section, we show that the two widespread ways of quantifying a rule’s productivity that we discussed above in Section Other Distributional Accounts of Rule Productivity—the Tolerance Principle (Yang, 2016) and potential productivity *P* (Baayen, 2001)—both fall short of capturing the behaviour we observed in the experiment.

#### The Tolerance Principle.

Above in Section An Urn Model of Rule Generalisation, we used the Tolerance Principle to inform one generalisation strategy that simulated learners might employ. We saw there that the Tolerance Principle strategy does not align with actual learners’ behaviour. In this section, we work out the maths to illustrate why.

In Group 1, participants learn *N* = 8 types in total (four with the Skew4 suffix and four with the Unif4 suffix). For *N* = 8, the threshold $\theta_{N}=\frac{8}{\ln8}\approx 3.8$. The Skew4 suffixing rule has four exceptions *e*: the four types that use the Unif4 suffix. And the Unif4 suffixing rule also has *e* = 4: the four types that use Skew4. Because *e* > 3.8, the Tolerance Principle suggests that neither the Skew4 or the Unif4 rules should be productive.

In Group 2, participants learn *N* = 12 types in total (four with Unif4, eight with Unif8). For *N* = 12, $\theta_{N}=\frac{12}{\ln12}\approx 4.8$. For the Unif4 suffixing rule in Group 2, *e* = 8. This exceeds the number of permitted exceptions, so the Unif4 rule should not be productive. But for the Unif8 suffixing rule, *e* = 4, below the threshold of 4.8.

All in all, the Tolerance Principle indicates that the Unif8 suffixing rule alone should be the productive one, an interesting contrast to our experiment results. Our participants’ preference for generalising with Unif8 over Unif4 does align with the Tolerance Principle’s predictions, but their preference for generalising with Skew4 over Unif4 does not. Further discussions of some limitations of the Tolerance Principle for measuring rule generalisability can be found in Hernandez et al. (2019) and Kapatsinski (2018).

#### Potential Productivity.

Potential productivity *P* represents the growth rate of the vocabulary (Baayen, 2001, 49–50): as one progresses token by token through a sample, how quickly does one encounter new types?

In more mathematical terms, the expression

$$P=\frac{\text{number}\;\text{of}\;\text{hapax}\;\text{legomena}\;\text{in}\;\text{the}\;\text{sample}}{\text{number}\;\text{of}\;\text{tokens}\;\text{in}\;\text{the}\;\text{sample}}$$

gives the slope of a tangent line to the vocabulary growth curve (like the ones shown in Figure 8) at the total number of tokens observed in the sample.

In spirit, *P* aligns well with our proposal, since it focuses on how many rare items appear in a sample. But it is limited by only counting hapax legomena, that is, types that appear only once.

Table 5 computes *P* for each distribution at the end of the first training block and after all three training blocks. (Because the number of tokens in each sample are equivalent, it is mathematically valid to compare values of *P* within each column; Evert & Lüdeling, 2001.) Based on *P*, only Skew4 stands a chance at being generalised, and only after the first block of training. After further training, since even the lowest-frequency word is encountered once per block, it will have been seen a total of three times, so *P* is always zero. (This is why we did not include *P* in the urn model simulations in Section An Urn Model of Rule Generalisation, since after 72 tokens, it would not weight the simulated learners’ decisions either way.)

**Table 5.** Potential productivity *P*, the number of hapax legomena out of the number of tokens, for each distribution observed in the experiment. Only the rule seen with Skew4 is predicted to be productive, and only after the first block of training. Once no more hapaxes are being encountered, none of the rules should be considered generalisable.

	After Block 1	After Block 3
Skew4	*P* = 1/24 = 0.04	*P* = 0/72 = 0
Unif4	*P* = 0/24 = 0	*P* = 0/72 = 0
Unif8	*P* = 0/24 = 0	*P* = 0/72 = 0

That *P* is non-zero only for small sample sizes underscores our suggestion above that learners’ generalisation behaviour should be tested after they’ve encountered different amounts of data. The rational Bayesian approach, in which both Skew4 and Unif8 should prompt generalisation after observing 24 tokens, could be contrasted with the *P* approach which suggests that only Skew4 should do so.

### Low Absolute Frequency or Low Relative Frequency?

A reviewer posed the question of whether we consider “low frequency” to mean low *relative* frequency (i.e., that some types are rarer than others) or low *absolute* frequency (i.e., that types appear with small frequency values of 1, 2, 3, etc.). In other words, do people generalise with Unif8 over Unif4, say, because Unif8 contains types with lower frequency than Unif4? Or because Unif8 contains types with frequency = 3, and that value is low enough to prompt generalisation?

We don’t have enough information yet to give a clear answer to this very interesting question. On the one hand, our instinct is that relative frequency is important: as long as one rule’s frequency distribution contains types that are rarer than some other rule’s, then that first rule should be more likely to be generalised. This instinct is behind how we implemented the low-frequency tail preference in the urn model in Section An Urn Model of Rule Generalisation. On the other hand, though, that reasoning might not hold up when looking at generalisation through the lens of Bayesian reasoning. In Bayesian terms, the uncertainty of learners’ representations is directly affected by absolute frequency. We saw this in the estimated population distributions in the Bayesian model in Section Generalisation as Rational Bayesian Inference: the larger the samples were, the narrower (and thus the less uncertain) the model’s posteriors. So absolute frequency should also influence the generalisation process. The interplay of relative and absolute frequency is an empirical question that certainly merits further research.

The same reviewer also wondered whether there would be a principled way to set a threshold on low absolute frequency, such that only types that appear sufficiently rarely would count as “low frequency” for the purpose of determining which rules should be generalised. This may be possible—it’s another empirical question—and such a threshold could be useful, for example, for incorporating a low-frequency preference into existing measures like the Tolerance Principle. However, the idea of a low frequency threshold doesn’t fit entirely comfortably into our view of rule generalisability. We think that a rule’s generalisability can best be understood as a gradient concept, not a binary one (Baayen, 2001; Bauer, 2001). And the danger of imposing a threshold on a gradient concept is that information gets lost, as nuance is compressed down to a binary decision.

### On the Emergence of Skewed Distributions in Language

Processes observed in experiments like ours can reflect the processes which, over evolutionary time, may shape language. This connection is at the core of empirical research on language evolution (e.g., Arnon & Kirby, 2024; Culbertson et al., 2020; Kirby et al., 2008, 2014; Smith, 2022), and in this penultimate section, we connect our present proposal to this broader picture.

We have shown that what feeds the process of rule generalisation is the presence of low-frequency items in a language user’s input. Now consider what kind of output is produced when a rule is generalised. Imagine that a language user is confronted with a novel stem and chooses to apply a given rule. That novel form then becomes a low-frequency form in their interlocutor’s input. Thus, through language use, rule generalisation produces low-frequency items—the very items that prompt people to generalise a rule in the first place. Based on this reasoning, we suggest that rule generalisation is a self-sustaining evolutionary process: it produces the same distributional structure which feeds it.

And, even more ambitiously, we suggest that perhaps it’s even by this mechanism that a population can arrive at relative grammatical convergence. If a whole population is exposed to comparable frequency distributions within a rule, then they are all more likely to generalise that rule. And if the rules that one person can use in a generalisable way are also the rules that someone else can use in that same way, then those two people can communicate and understand one another without hindrance.

However, there’s also a core aspect of natural language that our proposal does not account for. We’ve focused on an overarching explanation for previous findings which unifies certain properties of skewed and uniform distributions. But in natural human language, it’s skewed frequency distributions, not uniform ones, that are ubiquitous (Piantadosi, 2014; Zipf, 1949). With this unified account, we fail to explain why only one of these kinds of distributions is more common in language than the other.

We believe that many different processes, active at different levels of language and cognition, lead to language’s skewed frequencies, and that our account is only part of this broader picture. For example, on a very basic level, the frequency distributions of material objects are skewed (Clerkin et al., 2017; Lavi-Rotbain & Arnon, 2021; Long et al., 2021), so some skewed distributions in language may just reflect how frequent objects are in the world. But even without any reference to meaning, skewed distributions can aid the segmentation of continuous linguistic input into its component chunks (Kurumada et al., 2013; Lavi-Rotbain & Arnon, 2019a, 2019b, 2021, 2022). And through cultural transmission, units begin to follow a skewed distribution—whether because skew facilitates segmentation and learning (Arnon & Kirby, 2024), or through a process of purely random sampling (Keogh & Pankratz, 2024; Moscoso del Prado, 2013), or some of both.

We do not try here to weigh in on the interaction between all those other processes and the process of linguistic rule generalisation. Our contribution is to offer one explanation for why long-tailed frequency distributions are maintained in natural language: we suggest that, for as long as humans are generalising linguistic rules, our languages will contain distributions with low-frequency tails.

## CONCLUSION

In this paper, we investigated one source of information that language users draw on to decide whether they can generalise a rule: the distributional structure of the items that the rule has previously applied to. We built on studies that found facilitatory effects both of skewed frequency distributions and of distributions with a large type count. With an artificial language learning experiment, we replicated both of these results. And with an urn-based model of learning, we verified that the key property that informs how both kinds of distributions prompt generalisation is how many low-frequency types they contain. We suggest that this behaviour is consistent with learners applying rational Bayesian reasoning about how likely it is that a rule comes from a population larger than the sample they have already seen.

All in all, we suggest that rule generalisation is an example of a self-reinforcing evolutionary process in action. When a language user generalises a rule, the items they produce become low-frequency items in somebody else’s input, giving rise to the very property that helps others to generalise that rule.

## ACKNOWLEDGMENTS

We thank Lisa S. Pearl, Kenny Smith, Andy Wedel, Frederik Hartmann, and one anonymous reviewer for their immensely helpful comments on earlier versions of this work.

## FUNDING INFORMATION

EP’s work on this project was supported by the UK Economic and Social Research Council (award number ES/P000681/1) and the Social Sciences and Humanities Research Council of Canada (award number 752-2021-0366).

## Notes

^1^ The preregistration can be viewed at https://osf.io/5keh9/.^2^ We first piloted this study as a semantics-free statistical learning experiment using the serial reaction time (SRT) paradigm of Misyak et al. (2010a, 2010b). We needed participants to have learned the artificial language’s words very well, so that we could be confident that they would generalise based on their knowledge of the language. However, we found word learning in the SRT paradigm to be poor (as indeed Misyak and colleagues did as well). Changing the experimental format to incorporate arbitrary semantic mappings improved participants’ word learning substantially.^3^ We had preregistered an exploratory analysis of all participants who had “reliably” learned the language, a threshold we had set at 75% accuracy. This threshold appears to have been too conservative, since it was met or surpassed by 95 out of 100 participants, and the resulting analysis is effectively identical. We therefore present this exploratory analysis of only the perfect learners in the spirit of our preregistration.^4^ This framing bears some similarity to the dual-route model of morphological processing, the idea that language users have memorised all irregularly inflected forms and otherwise apply a default regular rule as the “elsewhere” case (see, e.g., Marcus et al., 1992; Pinker & Prince, 1988). The morphological structure of our artificial language, like that of English, does lend itself easily to such an analysis. But we wish to remain agnostic about how regular and irregular morphology is processed, especially considering the abundant evidence that dual-route processing does not make sense for many non-English morphological systems (see, e.g., Dabrowska, 2001; Ellis & Schmidt, 1998; Orsolini et al., 1998; Szagun, 2011).^5^ This idea is similar, we note, to the motivation behind Yang’s (2016) Tolerance Principle: to discover which rules should be generalised by minimising the average retrieval time.

## APPENDIX A: SPECIFICATION OF BAYESIAN LINEAR REGRESSION MODELS

The formula in R syntax for the model we fit to the learning data in Section Overall Learning Accuracy is correct ∼ distribution + (1|participant). The formula for the generalisation model in Section Generalisation is chose_non_baseline ∼ group +(1|participant). Both models share the same family and the same weakly regularising priors that we determined using prior predictive checks. The rest of both models’ specifications is the following: brm( family = bernoulli(link = logit), prior = c(  prior(normal(0, 1.5), class = Intercept),  prior(normal(0, 2), class = b),  prior(normal(0, 5), class = sd) ) )

The model that we used to estimate the association of centered log word frequency on correct responses (Section Learning Accuracy by Word Frequency) is the following: brm( correct ∼ logfreq_c + (1 + logfreq_c | ppt_id), family = bernoulli(link = logit), prior = c(  prior(normal(0, 1.5), class = Intercept),  prior(normal(0, 2), class = b),  prior(normal(0, 5), class = sd, coef = Intercept, group = ppt_id),  prior(normal(0, 5), class = sd, coef = logfreq_c, group = ppt_id),  prior(lkj(2), class = cor, group = ppt_id) ) )
